# Cortical thickness and functional connectivity changes in Chinese chess experts

**DOI:** 10.1371/journal.pone.0239822

**Published:** 2020-10-07

**Authors:** David J. Ouellette, Dan-Ling Hsu, Patricia Stefancin, Timothy Q. Duong

**Affiliations:** 1 Biomedical Engineering, Stony Brook University, Stony Brook, New York, United States of America; 2 Radiology, Stony Brook University, Stony Brook, New York, United States of America; University of Electronic Science and Technology of China, CHINA

## Abstract

**Background:**

Repeated practice to acquire expertise could result in the structural and functional changes in relevant brain circuits as a result of long-term potentiation, neurogenesis, glial genesis, and remodeling.

**Purpose:**

The goal of this study is to use surface-based morphology (SBM) to study cortical thickness differences in Chinese chess experts and novices, and to use regions of cortical thickness differences as seeds to guide a resting state connectivity analysis of the same population.

**Methods:**

A raw public dataset from Huaxi MR Research Center consisting of 29 Chinese chess experts and 29 novices was used in this study, with both T1-weighted and resting state functional MRI. Surface based morphometry was performed on the T1 images with the Freesurfur pipeline, with a vertex significance threshold of *p*<0.05 and a cluster false discovery rate of α < 0.05. Regions with significant differences were used in a seed-based comparison of resting state functional connectivity carried out with Statistical Parameter Mapping (SPM) and the Connectivity Toolbox (CONN). Regions of connectivity differences within groups were computed with a voxel significance threshold of *p*<0.05 and a cluster false discovery rate of α < 0.01.

**Results:**

Ten regions of the cortex of Chinese chess experts were found to be thinner than chess novices, including regions involved in visual processing, attention, working and episodic memory, and mental imagery, as well as several regions in the prefrontal cortex. There were no regions where experts’ cortices were thicker than novices. Three of the thinner regions exhibited increased functional connectivity to distant brain regions in chess experts.

**Conclusions:**

Brain regions that are structurally affected by chess training are associated with processes that would likely have a high utility in chess expertise. Using a hierarchical control model, we hypothesize that the functional changes linked with some of these structural changes are related to the professionally trained chess players’ ability to perceive and use contextual information, visuospatial perception, and outcome prediction in the domain of chess, all contributing to their exceptional performance.

## Introduction

The game of chess has long been used as a tool of cognitive science because it conveniently packages basic cognitive processes, such as memory, perception, and problem-solving [1]. It is also a pastime with a large number of very high performers, who often spend hours per day in chess practice. The amount of practice to acquire a specific expertise [2] may result in long-term potentiation, neurogenesis, glial genesis, and remodeling of different cellular and vascular components in the brain [3], resulting in regional structural and functional reorganization. Chess experts have been shown to have exceptional memories for board positions, including the ability to memorize and reproduce a complicated chess position in mere seconds and to play against a group of players simultaneously while blindfolded [4, 5]. In one early study of chess expertise, Dutch psychologist and chess master Adriaan De Groot asked a group of grand masters and less experienced (yet still expert) players to analyze a certain chess position and find the best next move. By listening to the players narrate their problem-solving process, De Groot determined that grand masters and lesser experts had the same depth of search: both groups considered an average of 3.5 moves ahead. The difference, De Groot found, was that grand masters were able to immediately understand the implications of the position and focus on promising paths, while less experienced players wasted time on dead-ends [4]. De Groot concluded that it is not necessarily superior reasoning that sets grand masters apart, but superior perception.

A few MRI studies have shed light on the neural underpinnings of the superior perception of chess experts. Functional MRI (fMRI) studies by Bilalic et. al on the visual expertise domains of chess and radiology show that repeated practice in visual tasks can lead to the recruitment of the fusiform face area (FFA) for domain-specific visual recognition [6, 7]. Beyond pure perception, other fMRI studies of chess have implicated brain regions associated with working memory and goal-based learning [8, 9]. Two studies reported a smaller caudate in chess experts compared to novices using voxel-based morphometry (VBM) [8, 9] and reduced regional cortical thickness using surface-based morphometry (SBM) in chess experts compared with novices [8]. However, there is still a lack of SBM studies on chess expertise, and none that examines the functional connectivity changes that accompany cortical thickness changes in experts.

The goal of current study was to identify regional cortical thickness differences in Chinese chess experts compared to novices, and to use those regions as seeds in the analysis of resting state fMRI (rsfMRI) data. This will help give greater context to what those structural changes might mean and how they relate to specific cognitive processes used by chess experts. This research will help add to our knowledge of expert performance in chess, an important “model” pursuit in cognitive science.

## Materials and methods

### Subjects

A raw public dataset from Huaxi MR Research Center [10] consisting of 29 Chinese chess experts (29±11yo; 9F) and 29 novices (26±7yo; 15F) matched for education level was used in this study. The criteria for expertise was set at the 2200 ELO rating cutoff with a training time of 4.24±1.73h/day). The novice group consisted of casual chess players with no significant ELO rating.

### MRI parameters

One whole-brain T1 weighted anatomical image (repetition time (TR) = 1900 ms; echo time (TE) = 2.26 ms; flip angle: 12°; voxel sizes = 1mm isotropic) and T2* weighted BOLD imaging (EPI, TR = 2000 ms; TE = 30ms; flip angle = 90°; voxel sizes = 3.75 x3.75 x 5 mm^3^; 205 volumes) were acquired for each subject using a standard clinical 3 Tesla MRI scanner [10].

### Structural processing

T1 weighted images were segmented and parcellated using Freesurfer [11] to extract cortical surface models for each subject. The automatic cortical segmentation was inspected visually and manually corrected when necessary. One expert subject was removed from the study due to poor Freesurfer segmentation. Cortical thickness maps were blurred with a 10mm Gaussian kernel and normalized to the average subject space. Cortical thickness was compared between experts and novices on a vertexwise basis with a two-tailed t-test with a significance threshold of p < 0.05 using Freesurfer’s GLMFit tool. A cluster size false discovery rate threshold of α < 0.05 was used to correct for multiple comparisons. Significant surface clusters were converted to volumes for seed-based connectivity analysis. Correlation analysis in the expert chess group was performed between cortical thickness and several behavioral metrics provided with the MRI data, while controlling for age: years of chess experience, ELO score, and hours per day of practice [10].

### rsfMRI processing

Resting state fMRI was processed using SPM12 and the Connectivity Toolbox (CONN). Subject scans were slice-timing corrected, then realigned and corrected for susceptibility-by-movement artifacts with SPM12’s “Realign & Unwarp” tool, realigning each time series to the first slice. Because the study population is entirely Chinese and the MNI template is based on Caucasian brains, a study-specific T1-weighted brain template was created and used for normalization using the BuildTemplateParallel script from the Advanced Normalization Tools (ANTs) package, run with default settings on all subjects [12]. This procedure has been shown to reduce the required deformations to normalize East Asian brains when compared to using an MNI target [13]. An atlas was created for the template using the Joint Label Fusion tool from ANTs with default settings on 20 pre-labeled brains from the Mindboggle-101 data set, specifically the OASIS-TRT-20 group [14, 15]. Agreement between this new study-specific template and the one used by Freesurfer for cortical thickness comparisons, as well as the atlas parcellation accuracy, was verified by visual inspection. Functional and anatomical images were resliced to 2x2x2mm voxels during normalization. The Artifact Removal Tools (ART) were used to scrub outlier volumes with excessive motion using moderate settings, removing 3% of the data. White matter and CSF masks were created by segmentation and thresholding the resulting tissue probability maps at 50%, and then performing one erosion step. The mean signal from the remaining masks was used as nuisance regressors to remove the white matter and CSF signal. Motion parameters, their derivatives, and their squares were also used as regressors. The time series was filtered with a .008-.09 Hz band-pass filter to remove physiological signals. Functional data was smoothed with an 8mm gaussian kernel and seed-based functional connectivity analysis was performed with seeds from the SBM findings. Voxelwise differences in connectivity were compared between groups with a voxel threshold of p<0.05 and with a cluster-size False Discovery Rate corrected p<0.01 to correct for multiple comparisons.

## Results

### Surfaced-based morphometry

**Fig 1:**
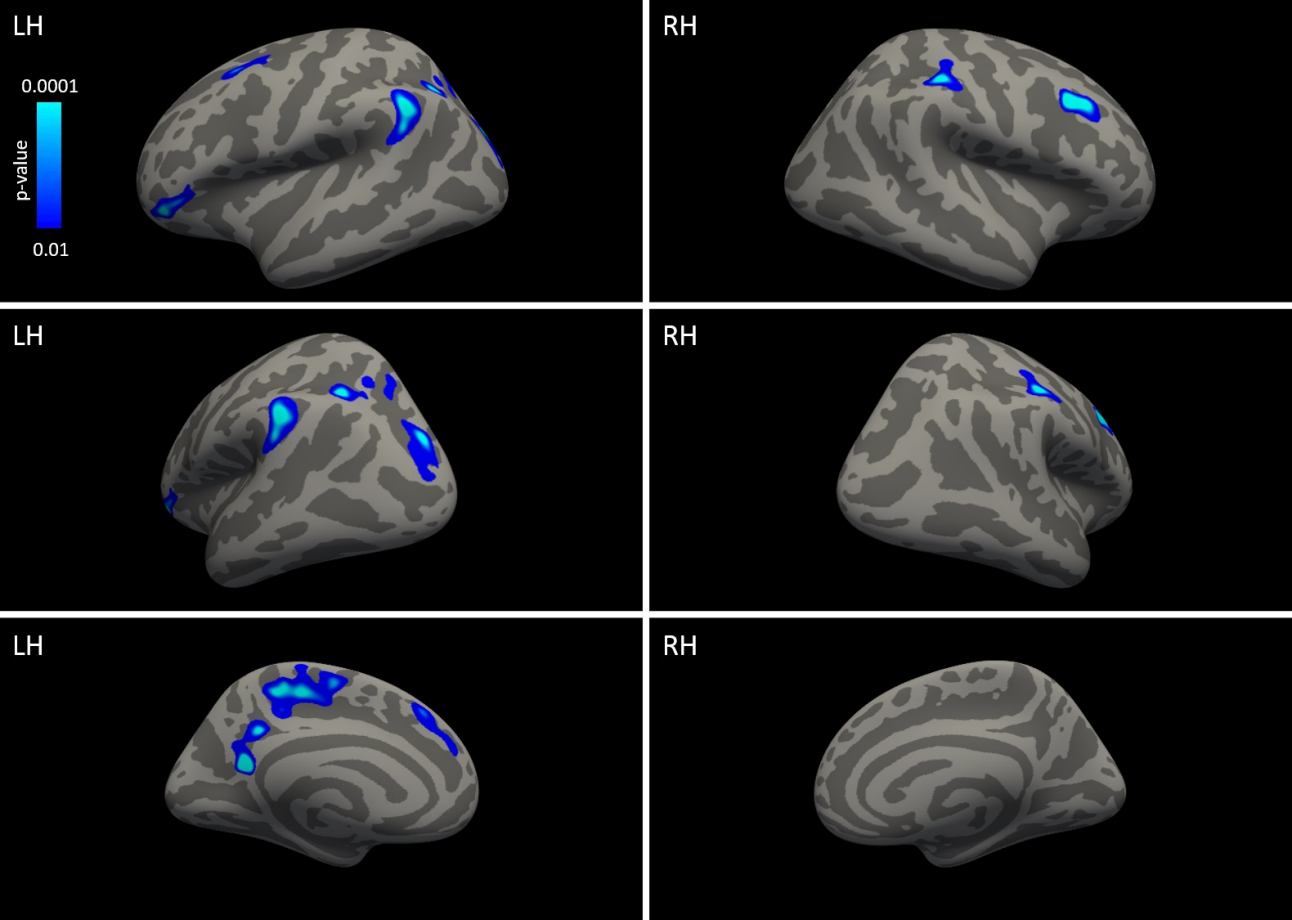
Inflated view of cortical thickness differences in Chinese chess experts.

Table 1 summarizes the ten regions where the cortex of expert Chinese chess players was significantly thinner than in novices. No regions where the cortex was significantly thicker in experts was identified. Fig 1 shows the significantly different clusters on an inflated gray matter surface. There was no significant correlation in chess experts between cortical thickness and ELO score, years of chess experience, or hours per day of chess practice.

**Table 1:** 

Region	MNI Center of Gravity	Size (voxels)	Cluster *p*-value	Functions
Right Caudal Middle Frontal	40, 22, 36	786	.0179	Orienting attention [16], episodic retrieval
Left Superior Parietal	-30, 64, 42	912	.0355	Spatial Orientation, goal intensive processing; visuomotor attention
Right Supramarginal	48, -27, 47	941	.0177	Visuospatial guidance; motor planning; deductive reasoning
Left Visual Association Area (V2)	-26, -87, 16	941	.0195	Visual mental imagery
Left Caudal Middle Frontal	-31, -8, 51	983	.0191	Inhibition; modulating attention
Left Supramarginal	-56, -49, 32	1289	.0080	Deductive reasoning; visuospatial guidance
Left Pars Orbitalis	-43, 41, -8	1318	.0070	Speech, parsing syntax
Left Superior Frontal	-6, 35, 38	1460	.0024	Spatially oriented processing, working memory
Left Precuneus	-7, -55, 23	1555	.0024	Visuospatial, memory, mental imagery; integration of information
Left Paracentral	-9, -29, 53	3240	.0002	Motor/sensory

### Seed-based connectivity

**Fig 2:**
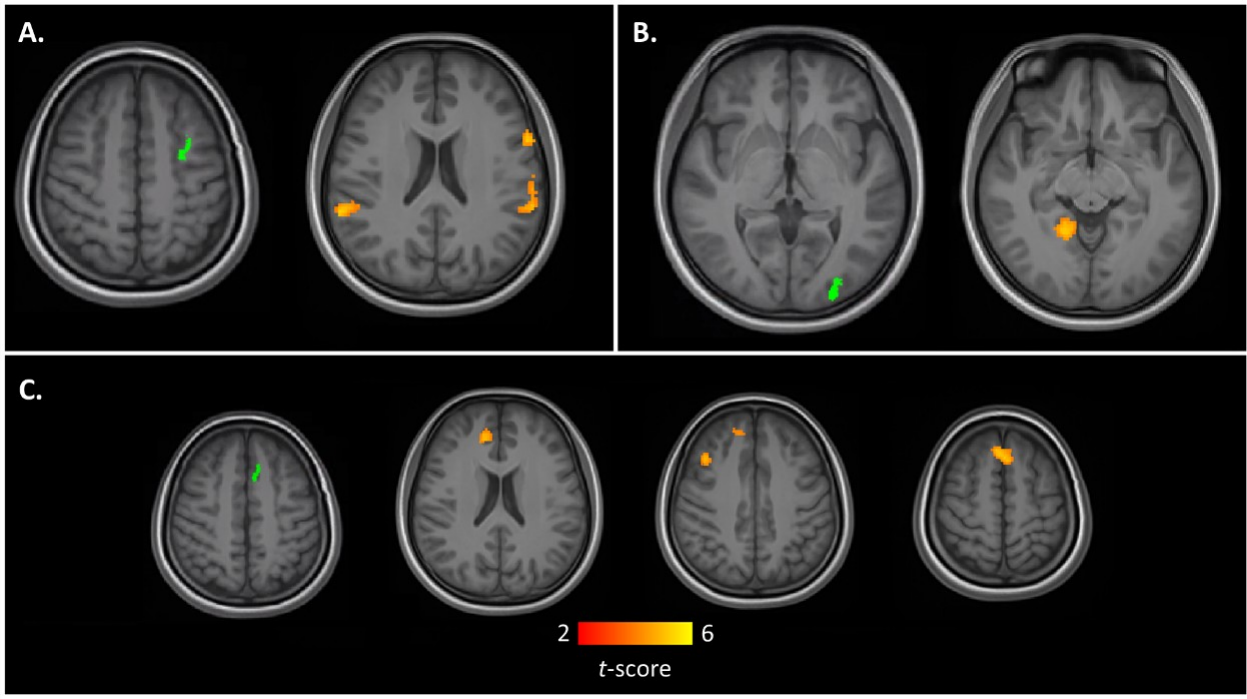
Increased resting state connectivity maps in expert chess players using cortical thickness seeds (in green) from A.) L. Caudal Middle Frontal gyrus, B.) L. V2, and C.) L. Superior Frontal Gyrus.

Three of the ten regions showed significant differences in functional connectivity between experts and novices. These regions are the left caudal middle frontal gyrus (MFG), left visual association cortex (V2), and left superior frontal gyrus (SFG) (Table 2, Fig 2).

**Table 2:** 

Seed	Clusters (x,y,z)	Size (voxels)	Region(s)	*t*-max	Size p-FDR
Left Caudal Middle Frontal	+60, -34, +32	233	Left Supramarginal	4.51	0.000012
	-58, -36 +28	197	Right Supramarginal	4.52	0.000028
	-58, -10, 16	80	Left Precentral	4.58	0.006612
			Left Pars Opercularis		
Left V2	+12, -46, 0	209	Right Lingual	4.90	0.000034
Left Superior Frontal	+2, +32, +46	121	Dorsomedial PFC	4.43	0.002288
	+12, +40, +14	106	Right ACC	4.51	0.002525
	+38, +24, +32	88	Right Rostral Middle Frontal	4.23	0.004583

## Discussion

### Cortical thickness

Cortical thickness analysis revealed ten cortical regions that were thinner in Chinese chess experts compared to novices. This study extended Duan et al.’s volumetric findings in which caudate volume was found to be smaller in Chinese chess experts compared to novices [9]. However, Duan et al. found that caudate volume was also negatively correlated with years of chess practice, controlled for age, while we did not detect any correlation between cortical thickness and chess practice. While it is possible that this indicates a difference in how training affects subcortical vs. cortical gray matter, it could simply be due to cortical thickness being a more variable parameter, making correlations difficult to detect.

Hänggi et al. used SBM to study experts of international chess and found cortical thinning in experts in the left supramarginal gyrus and the left precuneus [8], consistent with our study. They also found several other clusters of cortical thinning, but the only a cluster on the occipital temporal junction passed multiple comparison correction. We did not detect thinning in the occipital temporal junction as in our cohort. The discrepancy could be due to differences between Chinese and international chess or subject demographics.

Most of these 10 regions with reduced cortical thickness can be linked to processes that reflect the obvious mental demands of the game of chess: mental imagery, visuospatial processing, and working and episodic memory. However, there also seems to be a common thread of motor-related regions (motor planning, visuomotor attention) that are affected by chess training. It has been shown that physical actions can enhance performance in certain memory tasks [17, 18], and the question of the relationship between motor systems and higher cognition (sometimes called “motor cognition”) has recently gained interest [19]. It may be that the act of physically moving the pieces, or imagining them being moved, may play a role in what is traditionally thought of as a game of visual perception and cognition.

Three of the 10 regions with reduced cortical thickness have been reported to be involved in chess expertise using a game-like task during fMRI [20]: i) our left superior frontal gyrus overlaps with activation centered on the left anterior cingulate cortex (ACC) in Duan et al., ii) our left caudal middle frontal gyrus overlaps with Duan et al.’s peak activation in the left Frontal Eye Field (FEF), and iii) the precuneus was also found to be “deactivated” during game-like fMRI tasks. However, most areas of cortical thinning did not correspond to brain regions activated by the chess task. Thus, it appears that the thinner cortices of chess experts are not entirely tied to the brain regions used in a game-like situation.

The underlying mechanism of learning-based brain plasticity is not yet fully understood. Gray matter volume (GMV) changes have been reported even after short-term training. In a longitudinal study of GMV changes induced by a juggling task, GMV changes were detectible after only one week of training [19]. Driemeyer et. al concluded that these short-term volume changes are consistent with “fast adjusting neural systems, such as spine and synapse turnover”. Interestingly, Driemeyer et al. found that this change in volume did not correlate with task performance or amount of practice, conflicting with previous studies in chess experts which demonstrated an inverse correlation with years of chess experience and caudate size [4, 10]. Thus, the underlying mechanism behind the short-term GMV changes observed in the juggling study are unlikely to fully explain GMV decreases in chess experts. Hänggi et al. hypothesized that cortical thinning in international chess experts is due to increased neural efficiency and pruning in adolescence [10]. Since high-level chess experts usually begin training in childhood, it is possible that early adolescent neural pruning is modulated by this training, leading to thinner cortices in adulthood. In our and Hanggi’s study, the average age at the start of chess training was 8 years. Studying chess experts who start their careers in adulthood may help test this hypothesis.

### Functional connectivity

We identified three cortical regions with thinner cortex in the chess experts that had significantly increased connectivity to other areas of the brain when compared to controls. The left caudal middle frontal area has significantly higher functional connectivity to an area across precentral and the inferior frontal gyrus pars opercularis on the left hemisphere, and the bilateral supramarginal gyri. The left supramarginal gyri, along with is a hub of the left fronto-parietal network involved in language and memory tasks [21]. The left supramarginal area receives transmodal signals from nearby regions (e.g., visuospatial, audial, semantic, and somatosensory) [22]. Chess play involves manipulating chess pieces with different rules, developing strategies and modifying them according to context. According to Badre and Nee’s hierarchical control model of frontal cortex [23], the middle inferior frontal cortex is often involved in maintaining and updating the context, with information integrated and provided from the supramarginal region. The right supramarginal area, similarly, is also a hub of the right-lateralized fronto-parietal network whose response involves reasoning, attention, and inhibition [21]. Particularly, the right supramarginal gyrus has a role in empathy and theory of mind tasks [24]. It also has a role in motor planning of hand movements [25], which may or may not be related to chess expertise per se, but would certainly be used in a real-world chess match. Using Badre and Nee’s model, it is reasonable to speculate that long-term, intensive training of chess play can impose high demands on these functionally connected areas, propelling functional integration and reorganization of these areas to achieve more comprehensive contextual information.

The left visual association area (V2) in chess experts, which showed cortical thinning, has a higher functional connectivity to the right lingual gyrus. Both V2 and the lingual gyrus are visual processing areas. The lingual gyrus has been shown to be involved in visual memory, during both the initial encoding and subsequent recall of complex images [26]. Previous fMRI studies have also shown that the lingual gyrus is associated with visual attention, including searching for a specific object in a crowded visual field [27]. This strengthening of a visual-to-visual link in the chess expert is in line with the task-based experiment by Duan et al., which showed widespread visual activation [20], and with behavioral studies which have established the superior visual perception of chess experts [4, 5].

The seed from the left superior frontal gyrus (inside the dorsomedial prefrontal cortex) had stronger functional connectivity in expert chess players with three other prefrontal regions. The largest significant cluster extends from the seed region in the left DMPFC to the same region in the right hemisphere. There is also increased connectivity to a region on the right middle frontal gyrus within Brodmann area 9, which has been shown to be involved with visual spatial working memory [28]. Finally, there is increased functional connectivity from the left DMPFC to the right anterior cingulate cortex (ACC). According to Badre and Nee, the DMPFC is often coupled with the caudal supplementary motor area for contextual information and is sensitive to rewards and penalties, while working with the corresponding DLPFC according to the level of abstractness and the abruptness of the rewards and penalties. The more rostral a region is on the prefrontal cortex, the more likely it is to process more abstract and non-immediate rewards and penalties. Thus, the essence of DMPFC is to predict outcome with information provided by the lateral PFC [23]. Our results suggest a possibility that chess training involves training the DMPFC for enhanced predictions drawn from better functional connectivity with the rostral (BA10) and the more lateral (BA9) PFC, which provide schematic and contextual information respectively.

## Conclusion

We examined cortical thickness and the resting state functional connectivity associated with cortical thickness changes in Chinese chess experts and novices. We observed reduced cortical thickness in chess experts in areas responsible for visual processing, attention, working and episodic memory, and mental imagery, all processes that are naturally linked with the challenges of a game of chess. Moreover, we also discovered both cortical thinning and increased functional connectivity in several frontal areas. Due to the complexity of frontal functional organization, Badre and Nee’s hierarchical control model of frontal cortex provides a framework to explain these findings. With this model, we believe these structural and functional changes support the trained chess players’ ability to perceive and use contextual information, visuospatial perception, and outcome prediction in the domain of chess, all contributing to their exceptional performance. Aside from further uncovering the basis of high performance in chess, this finding may add to the already broad utility of chess as a tool in cognitive science, especially with regards to hierarchical control in problem-solving situations.
